# Development and Evaluation of Duplex MIRA-qPCR Assay for Simultaneous Detection of *Staphylococcus aureus* and non-*aureus* *Staphylococci*

**DOI:** 10.3390/microorganisms10091734

**Published:** 2022-08-27

**Authors:** Jiulian Lai, Zhenzhou Huang, Yue Xiao, Keyi Yu, Xuemei Bai, He Gao, Hang Dai, Xiaoning Liu, Duochun Wang

**Affiliations:** 1 Institution of Health Statistics and Epidemiology, School of Public Health, Lanzhou University, Lanzhou 730000, China; 2 National Institute for Communicable Disease Control and Prevention, Chinese Center for Disease Control and Prevention, State Key Laboratory of Infectious Disease Prevention and Control, Beijing 102206, China; 3 Center for Human Pathogenic Culture Collection, Chinese Center for Disease Control and Prevention, Beijing 102206, China

**Keywords:** *Staphylococcus aureus*, non-*aureus Staphylococci*, MIRA, duplex quantitative PCR, conventional PCR, detection

## Abstract

*Staphylococcus* spp., especially *Staphylococcus aureus* (*S. aureus*), is an important pathogen in hospital-acquired infection and food poisoning. Here, we developed a multienzyme isothermal rapid amplification combined with duplex quantitative PCR (duplex MIRA-qPCR) method, which can simultaneously detect the *S. aureus* species-specific conserved gene *FMN-bgsfp* and the *Staphylococcus* genus-specific conserved gene *tuf*. This assay enabled the amplification of DNA within 20 min at a constant temperature of 39 °C. Specificity analysis indicated that all nine common *Staphylococcus* species were positive and non-*Staphylococcus* spp. were negative for *tuf* gene, whereas *S. aureus* was positive, non-*aureus* *Staphylococci* species and non-*Staphylococcus* spp. were negative for *FMN-bgsfp* gene, suggesting that duplex MIRA-qPCR exhibited high specificity. Meanwhile, the sensitivity was tested and the limit of detection (LoD) was 3 × 10^2^ CFU/mL. The coefficient variation values ranged from 0.13% to 2.09%, indicating that the assay had good repeatability. Furthermore, all the nine common *Staphylococcus* species (including *S. aureus*) could be detected from four kinds of simulated samples and the LoD of *S. aureus* was 8.56 × 10^3^ CFU/mL. In conclusion, the duplex MIRA-qPCR has advantages of stronger specificity, lower detection threshold, shorter detection time, and simpler operation, which is an effective tool to detect *S. aureus* and non-*aureus* *Staphylococci* spp. infections rapidly.

## 1. Introduction

Members of the genus *Staphylococcus* are non-motile, facultative anaerobic, Gram-positive cocci that colonize on the surface of skin and the mucous membranes of humans and animals, including poultry [1]. *Staphylococci* are usually distributed in animals and the environment (such as soil and water, et al.) and can be differentiated into two groups according to the present or absent production of coagulase, i.e., coagulase-positive *Staphylococci* (CoPS) and coagulase-negative *Staphylococci* (CoNS) [2,3]. CoPS are usually assumed to be pathogenic, existing as commensals in humans and food-producing animals, and can cause severe or even lethal diseases [4]. *Staphylococcus aureus* (*S. aureus*) is the most common CoPS and regarded as the most virulent and notorious *Staphylococcus* species due to its large variety of expressed virulence factors [5]. The infection of *S. aureus* can cause bacteremia, sepsis, impetigo, atherosclerosis, necrotizing pneumonia, infective endocarditis, catheter endocarditis, osteomyelitis, pleuropulmonary infections and inflammation in skin, bone, joint and soft tissue [6,7]. Further, *S. aureus* is a frequent cause of infections acquired from hospitals and is one of the most common pathogens of prosthetic valve endocarditis [8]. In particular, the highly virulent methicillin-resistant *S. aureus* (MRSA) infection has a high mortality rate and leads to high treatment costs, which becomes a public-health threat around the world [9,10]. CoNS, however, have fewer virulence factors than the well-studied pathogen *S. aureus*, which generally colonize on human skin and contamination of blood cultures [11,12]. Although CoNS are widely considered to be less virulent with lower isolation frequency, they may also cause infection and are regarded as important opportunistic pathogens [13,14,15].

Therefore, there is an urgent need to establish a rapid, sensitive and reliable method to detect *S. aureus* and non-*aureus Staphylococci*. With the advancement of molecular biology detection technology, molecular methods, such as multilocus sequence typing (MLST), *Staphylococcus aureus* protein A (spa) typing, polymerase chain reaction (PCR) and quantitative PCR (qPCR), have been used to rapidly identify *Staphylococcus* spp. [16,17]. Although PCR and qPCR are some of the most valuable techniques currently used in bioscience, diagnostics and forensic science [18,19], there are certain disadvantages, such as cumbersome procedures, including electrophoresis and temperature change [20,21]. Multienzyme isothermal rapid amplification (MIRA), which is a novel nucleic acid rapid amplification technology, uses recombinant enzyme and primers to form a protein/single-stranded DNA complex (Rec/ssDNA). With the help of accessory proteins and single-stranded binding proteins (SSB), Rec/ssDNA invades the double-stranded DNA template and relies on the simultaneous activity of multiple functional proteins to rapidly amplify the target fragment. Due to its simple requirements for primers, high amplification efficiency and short detection time, MIRA has already been used to detect SARS-CoV-2 [22], hepatitis B virus [23] and *Mycobacterium tuberculosis* [24].

To this end, we developed a duplex MIRA-qPCR assay to detect *S. aureus* and non-*aureus Staphylococci* simultaneously. We first screened the *S. aureus* species-specific and *Staphylococcus* genus-specific conserved sequences from a public database and then designed primers and probes targeting the conserved sequences to evaluate the specificity, sensitivity and repeatability of this assay. At the same time, the detection of *Staphylococcus* was evaluated by using four common samples of *Staphylococcus*. Our results suggest that the duplex MIRA-qPCR can be applied in the detection of clinical treatment and food poisoning investigation.

## 2. Materials and Methods

### 2.1. Strain Information and DNA Extraction

In total, 61 strains, including 28 *Staphylococcus* spp. and 33 non-*Staphylococcus* spp. strains, were included in this study. All strains were identified at species level and stored at the Center for Human Pathogenic Culture Collection (CHPC), Chinese Center for Disease Control and Prevention. *Staphylococcus* spp. strains involved *S. aureus* (n = 8, including *S. aureus* ATCC 12600^T^) and non-*aureus Staphylococci* species (n = 20), namely, *S. epidermidis* (n = 3, including *S. epidermidis* ATCC 14990^T^), *S. haemolyticus* (n = 3, including *S. haemolyticus* ATCC 29970^T^), *S. saprophytics* (n = 3, including *S. saprophytics* ATCC 15305^T^), *S. xylosus* (n = 2), *S. caprae* (n = 3), *S. pasteuri* (n = 2), *S. pseudintermedius* (n = 2) and *S. hominis* (n = 2). Non-*Staphylococcus* spp. strains (n = 33) included 1 strain of *Listeria monocytogenes*, *Listeria ivanovii*, *Listeria innocua*, *Enterococcus faecalis*, *Enterococcus faecium*, *Enterobacter cloacae*, *Salmonella enterica serovar Typhimurium*, *Salmonella enterica serovar Enteritidis*, *Campylobacter jejuni*, *Campylobacter coli*, *Acinetobacter baumannii*, *Clostridium perfringens* and *Shigella sonnei*, 2 strains of *Vibrio parahaemolyticus*, 3 strains of *Klebsiella pneumonia*, 3 strains of *Vibrio cholerae*, 3 strains of *Shewanella* sp., 2 strains of *Aeromonas veronii*, 3 strains of *Escherichia coli* (including *Escherichia coli* O157 CHPC 1.1401), 2 strains of *Pseudomonas aeruginosa* and 2 strains of *Plesiomonas shigelloides*. Strains of *Campylobacter coli*, *Campylobacter jejuni* and *Clostridium perfringens* were inoculated on Columbia blood agar at 37 ± 0.5 °C for 24–72 h with 5% oxygen, 10% carbon dioxide (*Campylobacter coli* and *Campylobacter jejuni*) or no oxygen (*Clostridium perfringens*). Strains of *Listeria monocytogenes*, *Listeria ivanovii* and *Listeria innocua* were inoculated on Brain heart infusion agar at 37 ± 0.5 °C for 18–24 h. Strains of *Shewanella* sp. were inoculated on marine agar 2216E at 37 ± 0.5 °C for 18–24 h. Other strains were inoculated into Luria-Bertani (LB) broth at 37 ± 0.5 °C with shaking at 150 rpm for 8–10 h. Wizard Genomic DNA Extraction Kit (Promega, Madison, WI, USA) was used to extract DNA according to the manufacturer’s protocol.

### 2.2. Design of Primers and Probes

Publicly available genome data of over 18,000 *Staphylococcus* spp. isolates were retrieved from the National Center for Biotechnology Information (NCBI) assembly database, involving more than 60 *Staphylococcus* species. Taxonomic classification of *Staphylococcus* spp. was confirmed by type (strain) genome server (https://tygs.dsmz.de/) accessed on 20 July 2021 [25]. The core genes of *S. aureus* alone and all *Staphylococcus* spp. were identified by Roary pipeline (https://github.com/sanger-pathogens/Roary, accessed on 20 July 2021) with an identity cut-off of 50% [26]. Then, primers and probes were designed for the *S. aureus* species-specific conserved gene *FMN-bgsfp* (encoding FMN-binding glutamate synthase family protein) and *Staphylococcus* genus-specific conserved gene *tuf* (encoding elongation factor Tu) by Primer Premier 5 [27]. The specificity of primers was checked by basic local alignment search tool (BLAST) search (https://blast.ncbi.nlm.nih.gov/Blast.cgi, accessed on 20 July 2021). The quencher used was BHQ1 and the fluorophore used was FAM, which can be monitored in the qPCR system. The primers and probes were synthesized and purified by Sangon Bioengineering (Shanghai, China) Co., Ltd. The details of primers and probes sequences are shown in Table 1.

### 2.3. Conventional PCR and MIRA-qPCR

Conventional PCR amplification was performed by Labcycler PCR instrument (Sensoquest, Göttingen, Germany). The final volume of the amplification reaction mixture was 25 μL containing 12.5 μL of 2 × Es Taq MasterMix (Dye) (CWBIO, Beijing, China), 2 μL (10 μM) forward and reverse primers, 2 μL of genomic DNA and 8.5 μL of nuclease-free water. The amplification conditions comprised an initial 10 min denaturation step at 95 °C followed by 30 cycles of 10 s at 95 °C, 30 s at 56 °C and 30 s at 72 °C, with a final extension at 72 °C for 10 min. PCR products (3 μL for each sample) were electrophoresed through agarose gels (2%, w/v) at 80V for 50 min. Bands representing the corresponding fragment sizes were detected by Gel imager (Bio-Rad, California, USA).

MIRA-qPCR amplification was performed by Applied Biosystems QuantStudio 6 Flex Real-Time PCR (Singapore Life Technologies Holdings Pte Ltd., Singapore). MIRA basic kit (Amp-future, Weifang, China) was used for nucleic acid amplification according to the manufacturer’s instructions. MIRA-qPCR assay was performed in a 50 μL volume. The mixture was prepared in a tube containing 29.4 μL of Buffer A, 2 μL (10 μM) of forward primers, 2 μL (10 μM) of backward primers, 0.6 μL (10 μM) of probes, 9.5 μL of nuclease-free water and 4 μL of genomic DNA. When 2.5 μL of Buffer B was added into the 47.5 μL mixture, the reaction started. The amplification condition was at 39 °C for 20 min. The fluorescence values for FAM channel were collected every 30 s. It was determined negative if the threshold cycle (Ct) value was reported as undetermined with fluorescent signal maintained at background level [28].

### 2.4. Test for Specificity, Sensitivity and Repeatability

For the specificity test, MIRA-qPCR was performed with the genomic DNA templates from the 61 strains described above and conventional PCR was performed as control. The singular MIRA-qPCR was performed to verify the specificity of primers and probes for *S. aureus* (*FMN-bgsfp*) and *Staphylococcus* spp. (*tuf*), respectively. Then the duplex MIRA-qPCR was performed with specific primers and probes of *S. aureus* and non- *aureus Staphylococci.*

For the sensitivity test of MIRA-qPCR, the suspension of *S. aureus* ATCC 12600^T^ strain was 10-fold diluted. The concentrations of bacterial suspensions were calculated by plate counting. Wizard Genomic DNA Extraction Kit (Promega, Madison, WI, USA) was used to extract the genomic DNA from the diluted bacterial suspensions. Then the DNA templates were used for MIRA-qPCR assay. The limit of detection (LoD) of MIRA-qPCR was calculated based on the minimum concentration tested. Moreover, a comparative analysis of MIRA-qPCR and conventional PCR techniques was conducted.

For the repeatability test of MIRA-qPCR, three independent replicates were performed with the genomic DNA from the 10-fold serially diluted bacterial suspensions (the concentrations were 2.64 × 10^7^ CFU/mL, 3 × 10^7^ CFU/mL and 5.26 × 10^7^ CFU/mL, respectively) of *S. aureus* ATCC 12600^T^. The repeatability was evaluated by the coefficient of variation (CV,CV = SD/M × 100%) [29] of Ct values.

### 2.5. S. aureus and non-aureus Staphylococci Detection in Simulated Samples

The detection ability of MIRA-qPCR assay was further explored by four kinds of simulated samples (dried tofu, pork, milk and feces). Dried tofu and raw pork samples (each 40 g) were washed twice with 10 mL of sterile distilled water and then were dried inside the hood under ultraviolet light for 3 min for sterilization. Then, 40 g of dried tofu and raw pork was cut into about 2 g each piece and then homogenized in a stomacher (Stomacher Lab-Blender 400, Seward, London, UK) for 3 min, respectively [30,31]. Milk (5 mL) was centrifuged for 15 min (8000 rpm, 10 °C) to remove the upper cream layer. The remainder was filtered with 0.45 μm filter membrane to remove impurities. The filtered milk was then diluted with distilled water at a ratio of 1:20 [32]. Feces (2 g) were thoroughly suspended in 10 mL Tris-HCl buffer (20 mM, pH 7.8) [33]. The dried tofu, pork, milk and fecal samples were confirmed to be *Staphylococcus*-negative by conventional PCR [34].

Secondly, *S. aureus* ATCC 12600^T^, *S. epidermidis* ATCC 14990^T^, *S. haemolyticus* ATCC 29970^T^, *S. saprophyticus* ATCC 15305^T^, *S. xylosus* CHPC 1.3379, *S. caprae* CHPC 1.7854, *S. pasteuri* CHPC 1.7875, *S. pseudintermedius* CHPC 1.7919 and *S. hominis* CHPC 1.7929 strains were, respectively, inoculated into LB broth at 37 ± 0.5 °C with shaking at 150 rpm for 8 h. Then the bacterial suspensions of the above strains were added into saline solution containing dried tofu, pork, milk or feces to make simulated samples.

Thirdly, to test the LoD of MIRA-qPCR, the bacterial suspensions (approximately 8.56 × 10^7^ CFU/mL) of *S. aureus* ATCC 12600^T^ were 10-fold serially diluted. Then, normal saline solution containing dried tofu, pork, milk or feces was added into the bacterial suspension with a volume ratio of 1:1 to make simulated samples. The nucleic acids of the above strains were extracted from each stimulated sample by Wizard Genomic DNA Extraction Kit (Promega, Madison, WI, USA), then MIRA-qPCR assay was performed. Blank samples were used as negative controls. The LoD was evaluated as mentioned.

## 3. Results

### 3.1. The Principle and Workflow of Duplex MIRA-qPCR Assay

**Figure 1:**
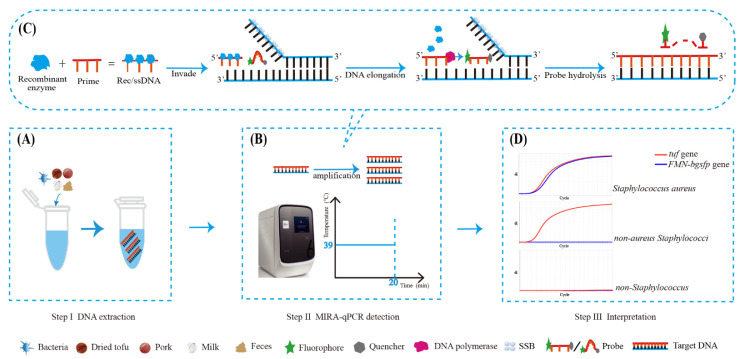
Schematic illustration of the workflow of duplex MIRA-qPCR assay. The detection process can be roughly divided into three steps. (**A**) Step I, the extraction of DNA. (**B**,**C**) Step II, the amplification and detection of DNA by MIRA-qPCR. (**D**) Step III, the interpretation of fluorescent signals.

This study aims to develop a novel strategy for the rapid and duplexed quantification of *S. aureus* and non-aureus *Staphylococci* by combining MIRA with qPCR. The target bacteria and samples were pretreated and DNA was extracted as the substrate for the next step (Figure 1A). The MIRA-qPCR enabled the amplification of DNA within 20 min at a constant temperature of 39 °C (Figure 1B). The detection principle of MIRA-qPCR is illustrated in Figure 1C. Recombinant enzyme and primers form a Rec/ssDNA. The SSB assists Rec/ssDNA to invade the double-stranded DNA template. When the complex Rec/ssDNA decomposes, the DNA polymerase binds to the 3’ end of the primer to start the chain extension. Finally, the probe is hydrolyzed and the target DNA is amplified. The appearance of fluorescence amplification curves of both *tuf* and *FMN-bgsfp* genes indicated the presence of *S. aureus* (Figure 1D). The appearance of a fluorescence amplification curve for only *tuf* gene but not *FMN-bgsfp* gene indicated the presence of non-*aureus* *Staphylococci* species. The absence of fluorescence amplification curves of *tuf* and *FMN-bgsfp* genes indicated the absence of any *Staphylococcus* species.

### 3.2. Specificity Analysis of the MIRA-qPCR

**Figure 2:**
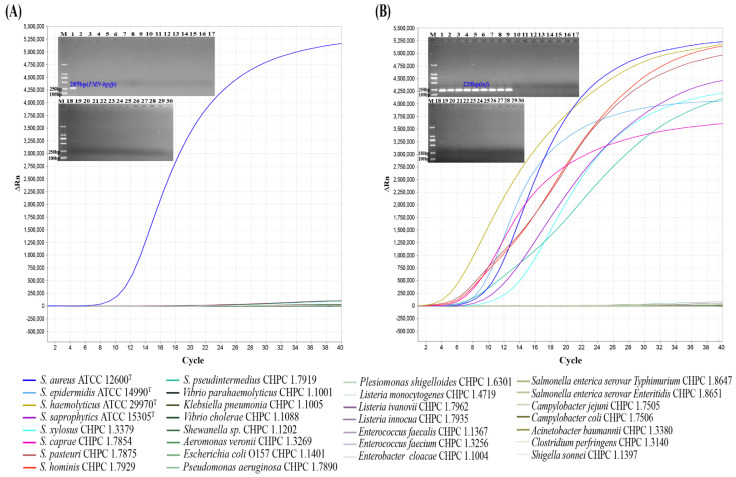
The amplification curves and the results of agarose gel electrophoresis of singular MIRA-qPCR and singular conventional PCR. (**A**) Detection of *S. aureus* species-specific gene *FMN-bgsfp*. (**B**) Detection of *Staphylococcus* genus-specific gene *tuf.* Insets show the results of conventional PCR. M: DL2000 Marker; Lane 1: *S. aureus* ATCC 12600^T^; Lane 2-9: *S. epidermidis* ATCC 14990^T^, *S. haemolyticus* ATCC 29970^T^, *S. saprophyticus* ATCC 15305^T^, *S. xylosus* CHPC 1.3379, *S. caprae* CHPC 1.7854, *S. pasteuri* CHPC 1.7875, *S. pseudintermedius* CHPC 1.7919 and *S. hominis* CHPC 1.7929; Lane 10-30: *Vibrio parahaemolyticus* CHPC 1.1001, *Klebsiella pneumonia* CHPC 1.1005, *Vibrio cholerae* CHPC 1.1088, *Shewanella* sp. CHPC 1.1202, *Aeromonas veronii* CHPC 1.3269, *Escherichia coli* O157 CHPC 1.1401, *Pseudomonas aeruginosa* CHPC 1.7890, *Plesiomonas shigelloides* CHPC 1.6301, *Listeria monocytogenes* CHPC 1.4719, *Listeria ivanovii* CHPC 1.7962, *Listeria innocua* CHPC 1.7935, *Enterococcus faecalis* CHPC 1.1367, *Enterococcus faecium* CHPC 1.3256, *Enterobacter cloacae* CHPC 1.1004, *Salmonella enterica serovar Typhimurium* CHPC 1.8647, *Salmonella enterica serovar Enteritidis* CHPC 1.8651, *Campylobacter jejuni* CHPC 1.7505, *Campylobacter coli* CHPC 1.7506, *Acinetobacter baumannii* CHPC 1.3380, *Clostridium perfringens* CHPC 1.3140 and *Shigella sonnei* CHPC 1.1397.

Nine *Staphylococcus* species (including *S. aureus*) and twenty-one non-*Staphylococcus* species were selected for the specificity analysis of MIRA-qPCR. Conventional PCR was performed as a control. In singular MIRA-qPCR, for the detection of *S. aureus* species-specific gene *FMN-bgsfp*, only *S. aureus* showed positive results in both MIRA-qPCR and conventional PCR, whereas the eight strains of non-*aureus Staphylococci* species and the twenty-one non-*Staphylococcus* strains showed negative results (Figure 2A). Similarly, for the detection of *Staphylococcus* genus-specific gene *tuf*, all nine strains of *Staphylococcus* species showed positive results in both MIRA-qPCR and conventional PCR, while all twenty-one non-*Staphylococcus* species strains showed negative results (Figure 2B). The same results were achieved using the other 31 strains of *Staphylococcus* species and non-*Staphylococcus* spp. strains (data not shown). These results showed that the primers and probes had strong specificity for both *S. aureus* and *Staphylococcus* spp.

**Figure 3:**
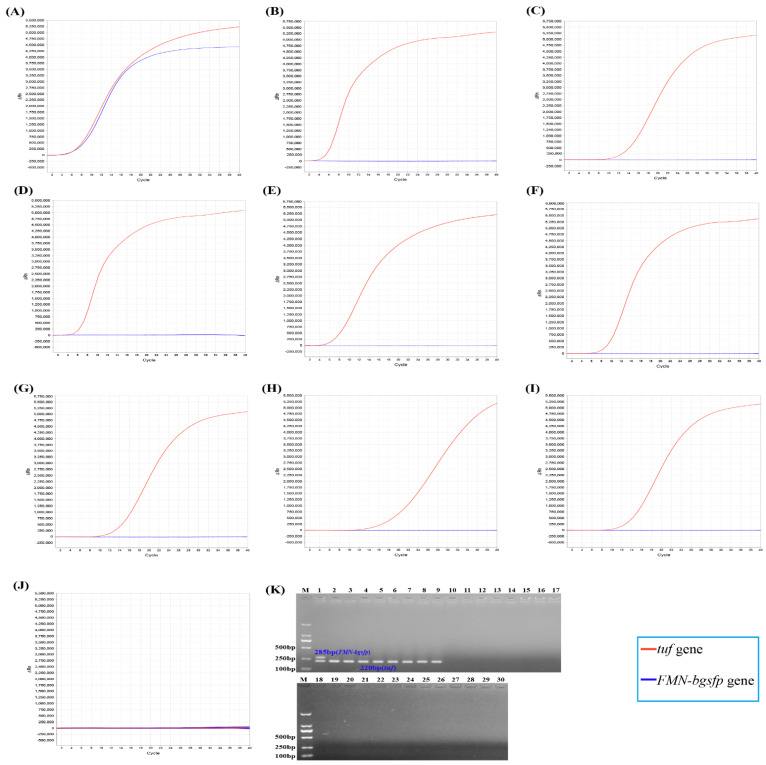
The amplification curves and the results of agarose gel electrophoresis of duplex MIRA-qPCR and duplex conventional PCR. (**A**) *S. aureus* ATCC 12600^T^; (**B**) *S. epidermidis* ATCC 14990^T^; (**C**) *S. haemolyticus* ATCC 29970^T^; (**D**) *S. saprophyticus* ATCC 15305^T^; (**E**) *S. xylosus* CHPC 1.3379; (**F**) *S. caprae* CHPC 1.7854; (**G**) *S. pasteuri* CHPC 1.7875; (**H**) *S. pseudintermedius* CHPC 1.7919; (**I**) *S. hominis* CHPC 1.7929 and (**J**) *Vibrio parahaemolyticus* CHPC 1.1001, *Klebsiella pneumonia* CHPC 1.1005, *Vibrio cholera* CHPC 1.1088, *Shewanella* sp. CHPC 1.1202, *Aeromonas veronii* CHPC 1.3269, *Escherichia coli* O157 CHPC 1.1401, *Pseudomonas aeruginosa* CHPC 1.7890, *Plesiomonas shigelloides* CHPC 1.6301, *Listeria monocytogenes* CHPC 1.4719, *Listeria ivanovii* CHPC 1.7962, *Listeria innocua* CHPC 1.7935, *Enterococcus faecalis* CHPC 1.1367, *Enterococcus faecium* CHPC 1.3256, *Enterobacter cloacae* CHPC 1.1004, *Salmonella enterica serovar Typhimurium* CHPC 1.8647, *Salmonella enterica serovar Enteritidis* CHPC 1.8651, *Campylobacter jejuni* CHPC 1.7505, *Campylobacter coli* CHPC 1.7506, *Acinetobacter baumannii* CHPC 1.3380, *Clostridium perfringens* CHPC 1.3140 and *Shigella sonnei* CHPC 1.1397. (**K**) The gel electrophoresis results of conventional PCR. M: DL2000 Marker; Lane 1: *S. aureus* ATCC 12600^T^; Lane 2-9: *S. epidermidis* ATCC 14990^T^, *S. haemolyticus* ATCC 29970^T^, *S. saprophyticus* ATCC 15305^T^, *S. xylosus* CHPC 1.3379, *S. caprae* CHPC 1.7854, *S. pasteuri* CHPC 1.7875, *S. pseudintermedius* CHPC 1.7919 and *S. hominis* CHPC 1.7929; Lane 10-30: *Vibrio parahaemolyticus* CHPC 1.1001, *Klebsiella pneumonia* CHPC 1.1005, *Vibrio cholera* CHPC 1.1088, *Shewanella* sp. CHPC 1.1202, *Aeromonas veronii* CHPC 1.3269, *Escherichia coli* O157 CHPC 1.1401, *Pseudomonas aeruginosa* CHPC 1.7890, *Plesiomonas shigelloides* CHPC 1.6301, *Listeria monocytogenes* CHPC 1.4719, *Listeria ivanovii* CHPC 1.7962, *Listeria innocua* CHPC 1.7935, *Enterococcus faecalis* CHPC 1.1367, *Enterococcus faecium* CHPC 1.3256, *Enterobacter cloacae* CHPC 1.1004, *Salmonella enterica serovar Typhimurium* CHPC 1.8647, *Salmonella enterica serovar Enteritidis* CHPC 1.8651, *Campylobacter jejuni* CHPC 1.7505, *Campylobacter coli* CHPC 1.7506, *Acinetobacter baumannii* CHPC 1.3380, *Clostridium perfringens* CHPC 1.3140 and *Shigella sonnei* CHPC 1.1397.

In duplex MIRA-qPCR, the fluorescence amplification curves of both the *FMN-bgsfp* gene and *tuf* gene were detected in the reaction of *S. aureus* (Figure 3A). The fluorescence amplification curves of *tuf* gene but not *FMN-bgsfp* gene were detected in the reactions of non-*aureus Staphylococci* species (Figure 3B–I). No fluorescence amplification curves were detected in the reactions of non-*Staphylococcus* spp. strains (Figure 3J). In conventional PCR, the same results were observed (Figure 3K). Our results showed that the duplex MIRA-qPCR exhibited high specificity for simultaneous detection of *S. aureus* and non-*aureus Staphylococci* strains.

### 3.3. Sensitivity Analysis of the MIRA-qPCR

**Figure 4:**
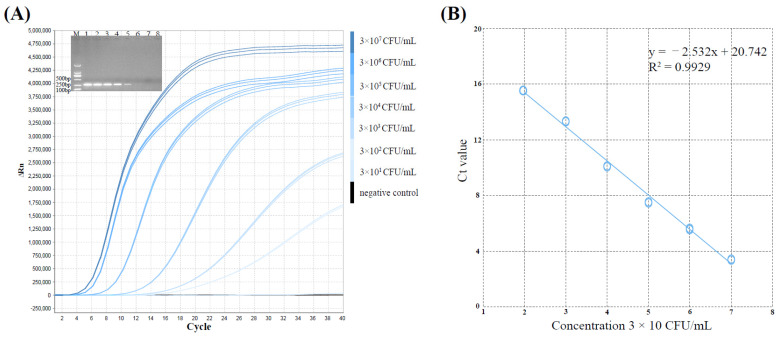
The LoD-relevant results of *S. aureus* ATCC 12600^T^ by MIRA-qPCR and conventional PCR. (**A**) The amplification curves showed the LoD of MIRA-qPCR and the inset showed the LoD of conventional PCR. M: DL2000 Marker; Lane 1-7: the corresponding concentration of bacterial suspensions is 3 × 10^8^~10^2^ CFU/mL; Lane 8: Negative control. (**B**) The standard curve between the Ct values and the concentrations of bacterial suspension.

The sensitivity of MIRA-qPCR assay was tested by the bacterial suspensions of *S. aureus* ATCC12600^T^ with serially diluted concentrations (3 × 10^8^~3 × 10^1^ CFU/mL) and all tests were repeated three times. The LoD of the MIRA-qPCR and conventional PCR assay with pure culture was 3 × 10^2^ CFU/mL and 3 × 10^4^ CFU/mL, respectively (Figure 4A). The LoD of the MIRA-qPCR was 100-fold more sensitive than that of conventional PCR. The linear regression analysis between Ct values and the concentrations of bacterial suspensions yielded R^2^ value above 0.99 and the slope was −2.532 (Figure 4B), indicating high linear relationship.

### 3.4. Repeatability Analysis of the MIRA-qPCR

In order to analyze the repeatability of MIRA-qPCR, three independent replicates were performed and the corresponding Ct values of fluorescence amplification curves were statistically analyzed. The CV values ranged from 0.13% to 2.09%, indicating good repeatability (Table 2).

### 3.5. Evaluation of MIRA-qPCR Assay Using Simulated Samples

**Figure 5:**
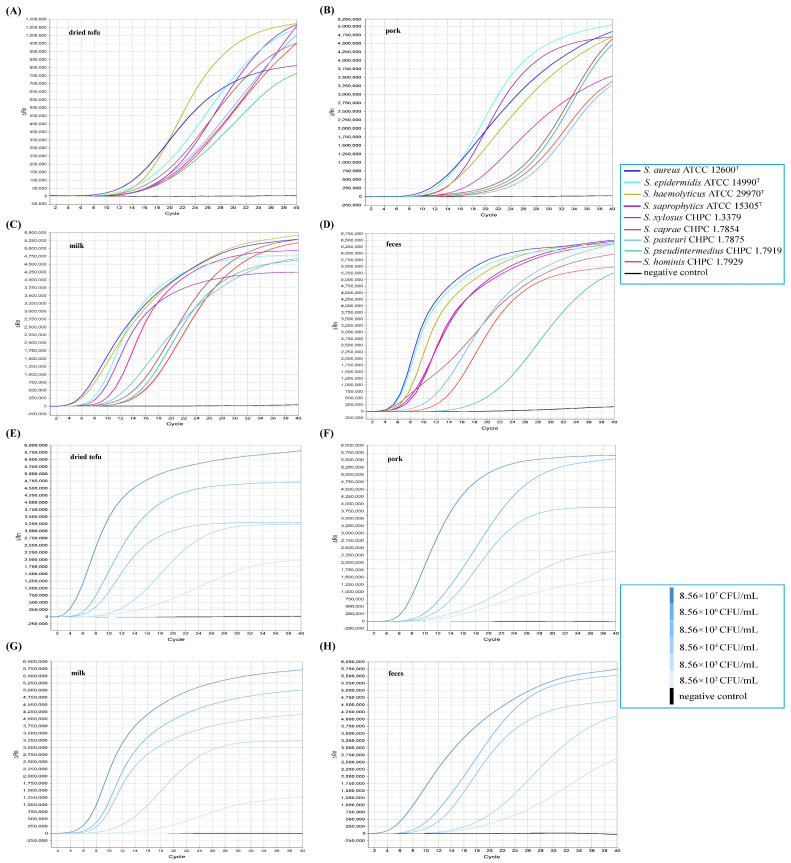
Detection of the *Staphylococci* strains in simulated samples by MIRA-qPCR. (**A**–**D**) Detection of nine *Staphylococci* species strains. (**E**–**H**) The LoD of *S. aureus* ATCC 12600^T^ strain.

To ascertain the applicability of the MIRA-qPCR assay as a surveillance tool for *Staphylococcus* spp., MIRA-qPCR assay was tested by artificially inoculating *S. aureus*, *S. epidermidis*, *S. haemolyticus*, *S. saprophytics*, *S. xylosus*, *S. caprae*, *S. pasteuri*, *S. pseudintermedius* and *S. hominis* stains into dried tofu, pork, milk and feces, respectively. The results showed that nine *Staphylococcus* species (including *S. aureus*) were successfully detected in the four simulated samples (Figure 5A–D). Additionally, for *S. aureus*, positive amplification can be observed when the concentrations of bacteria were more than 8.56 × 10^3^ CFU/mL (Figure 5E–H). No amplification curve was observed in simulated samples without *Staphylococcus*.

## 4. Discussion

Among *Staphylococci*, *S. aureus* is the most frequently isolated and the most important pathogen, but the incidences of non-*aureus Staphylococci* infection have increased throughout the world [8,35]. Therefore, it is most important to distinguish *S. aureus* from non-*aureus Staphylococci* in clinical practice and food safety investigation. It is also important to confirm the presence of non-*aureus Staphylococci,* given its increasing significance [14]. However, the detection of *Staphylococcus* mainly focused on certain species, especially *S. aureus* [36,37,38], and few studies detected *S. aureus* and *non-aureus Staphylococci* at the same time. Okolie et al. and McClure et al. [39,40] established multiplex conventional PCR assays detecting *S. aureus* and other *Staphylococci* spp. simultaneously. However, the complex thermal cycles, long detection time and low sensitivity limit its application. Therefore, our study aimed at proffering a new solution, MIRA-qPCR, to overcome the above limitations and make the detection simpler and more efficient.

The selection of targeted genes is critical for pathogenic bacteria detection. The *tuf* gene has been reported as a good target to allow the detection of *Staphylococci* at the genus level [41]. Sakai et al. developed a PCR-based assay that targets *tuf* gene for the identification of *S. aureus* and a variety of CoNS [42]. As for the identification of *S. aureus* species, the 16S rRNA, *nuc* gene and *femA* gene were used as target genes [36,40,43]. However, some of these genes cannot discriminate between phylogenetically closely related species, such as the *S. epidermidis* and *S. aureus* cluster groups [38]. Further, some researchers reported that some of these genes were unsuitable to detect poly-microbial samples harboring *S. aureus* and CoNS [39]. Rapid development of high-throughput sequencing technology has increased the number of whole-genome sequences available in public databases, making it easy to obtain novel target genes specific to *Staphylococcus* spp. using bioinformatics approaches [44]. Thus, we designed specific primers and probes of two conserved genes (*FMN-bgsfp* and *tuf*) for *S. aureus* species and *Staphylococcus* genus, respectively. MIRA-qPCR assay confirmed the specificity of these primers and probes and positive results were generated for *S. aureus* and non-*aureus Staphylococci* strains but not non-*Staphylococcus* strains.

MIRA-qPCR also exhibited high sensitivity. Our results showed that in conventional PCR, the LoD was 3 × 10^4^ CFU/mL, while in MIRA-qPCR system, the LoD was 3 × 10^2^ CFU/mL, which was 100-fold more sensitive than that of the conventional PCR assay. Wu et al. detected *S. aureus* based on loop-mediated isothermal amplification (LAMP), which showed high sensitivity with a detection limit of 4 × 10^2^ CFU/mL, similar to our results [45]. Furthermore, our statistical analysis of the Ct values showed that MIRA-qPCR had good repeatability. To evaluate the practical application of MIRA-qPCR to detect *S. aureus* and non-*aureus Staphylococci* strains, artificially contaminated dried tofu, pork, milk and fecal samples were analyzed. Positive amplifications of nine *Staphylococcus* species were observed in these four stimulated samples. The LoD of MIRA-qPCR was 8.56 × 10^3^ CFU/mL in simulated samples, which was approximately 10-fold less sensitive than that in pure bacterial suspension. Previously, some researchers reported that lower detection sensitivity for artificially contaminated samples was attributed to certain factors, such as food particles, nutrient content, pH value and inhibitors [46]. Thus, we assumed that simulated samples were negative at a bacterial concentration of 8.56 × 10^2^ CFU/mL in this study, which might be related to food and fecal particles or other potential factors.

In recent years, emerging isothermal amplification techniques have provided new opportunities for pathogen detection. Helicase-dependent amplification (HDA) was performed at 60~65 °C for 60~120 min [47,48]. LAMP has the advantages of accuracy and sensitivity [49], but it has some drawbacks, such as false-positive results [50]. Crossing priming amplification (CPA) with amplification temperatures at 37~42 °C for 20~40 min is efficient but is not ideal for discriminating between mutations or identifying mutations based on the performance of a nested recombinase polymerase amplification (RPA)-based assay [51]. Compared to the assays mentioned above, MIRA-qPCR has its own advantages in pathogen detection. Its detection time can be shortened to 20 min because of the high amplification efficiency of multienzyme cooperation. The amplification process is completed in a single run, which was unnecessary to open the lid in the process. Completely closed tube operation can greatly reduce the risk of contamination and improve the specificity of detection [52]. Moreover, the results of MIRA-qPCR assay can be interpreted by the fluorescence curves, which is simple and user friendly. Compared with conventional PCR and LAMP, agarose gel electrophoresis or turbidimetry is not required in MIRA-qPCR, which shortens the workflow and decreases the risk of contamination [53,54,55]. The duplex MIRA-qPCR assay in this study required only two pairs of primers for the detection of *S. aureus* and non-*aureus Staphylococci.* In LAMP, multiple primers were required, including two outer primers, two inner primers and two loop primers [56]. Simper primer requirements for MIRA-qPCR can avoid nonspecific amplification of DNA templates. In addition, the reagents used in MIRA-qPCR can be easily transported as freeze-dried powder. Primers and probes can be freeze-dried together with the reagents into commercial kits, which are easy and flexible for storage and, thus, are suitable for on-site real-time detection. In addition, MIAR-qPCR is cost effective with simple laboratory settings for analysis and only requires heating the samples to reaction temperature to accomplish rapid, sensitive and reliable detection. It is believed that as the costs further decrease in the future, MIRA-qPCR can gain wider application. 

However, the MIRA-qPCR assay had some limitations to overcome. Firstly, among *Staphylococci*, MRSA infections are common in hospital settings [57]. In addition to the genus of *Staphylococcus*, the common clinical and food-borne infection sources include many other bacteria, such as *Enterococcus faecalis*, *Enterobacter cloacae*, *Clostridium perfringens*, *Listeria monocytogenes* and *Campylobacter jejuni* [58,59,60,61].Therefore, the multiplex MIRA-qPCR for detecting MRSA, *Staphylococcus* spp. and bacterial pathogens of other genera can be established in the future. Secondly, the reaction temperature in MIRA-qPCR is close to room temperature, which means that the reaction is likely to start before the mixture is placed on the qPCR instrument. In that case, complete amplification curves cannot be seen and results cannot be interpreted. Further studies are needed to avoid this circumstance and to optimize the amplification temperature of MIRA-qPCR. Thirdly, a DNA extraction process is still required in MIRA-qPCR. The combination of DNA extraction and amplification can greatly increase the efficiency and convenience of MIRA-qPCR.

The MIRA-qPCR method is rapid, quantitative and feasible. The whole process can be accomplished within 20 min, which is far more efficient than the traditional process. Results showed that the duplex MIRA-qPCR exhibited strong specificity and high sensitivity and the coefficient variation revealed the assay had good repeatability. Moreover, the detection of simulated samples demonstrated good applicability of the as-developed assay. In summary, the MIRA-qPCR method provides a novel strategy for the simultaneous duplex detection of clinical and food-borne pathogens and has potential as a primary screening tool.

## Figures and Tables

**Table 1:** Primers and probes information.

Gene	Target	Sequence (5′–3′)	Product Sizes (bp)
*FMN-bgsfp*	*Staphylococcus aureus*	F: ATGACCAGCTTCGGTACTACTAAAGATTATC	285
		R: TCCATAACTCATACCAGATTGTCCTACGATAC	
		Probe:TCGCGAATGAGCGTTTATTTAGTCGTGAAGAATA[dT-FAM][THF]G[dT-BHQ1]GTACCGACAAAGATT[c3spacer]	
*tuf*	*Staphylococcus* spp.	F: CTTCCCAGGTGAYGAYGTACCTGTAATCGC	220
		R: ACCACGTTCAACACGGCCWGTAGCAACAGT	
		Probe: AACCATTCATGATGCCWGTTGAGGACGTAT[FAM-dT][THF][ BHQ1-dT]CAATCACTGGTCGTG [c3spacer]	

**Table 2:** CV values and Ct values of different replicates.

	Bacterial Concentration (CFU/mL)					
	N × 10^7^	N × 10^6^	N × 10^5^	N × 10^4^	N × 10^3^	N × 10^2^
Ct values for Replicate 1 (N = 2.64)	3.84	5.62	7.48	10.24	13.46	15.86
Ct values for Replicate 2 (N = 3)	3.75	5.68	7.43	10.17	13.42	15.82
Ct values for Replicate 3 (N = 5.26)	3.91	5.71	7.51	10.20	13.48	15.85
Mean of Ct values	3.83	5.67	7.47	10.20	13.45	15.84
SD of Ct values	0.08	0.05	0.04	0.04	0.03	0.02
CV (%)	2.09	0.88	0.54	0.39	0.22	0.13

## Data Availability

The original contributions presented in the study are included in the article. Further inquiries can be directed to the corresponding authors.
